# Spinal cord stimulation in Parkinson’s disease: a review of the preclinical and clinical data and future prospects

**DOI:** 10.1186/s42234-020-00041-9

**Published:** 2020-03-16

**Authors:** Yi Cai, Rajiv D. Reddy, Vishal Varshney, Krishnan V. Chakravarthy

**Affiliations:** 1 Department of Anesthesiology and Pain Medicine, University of California San Diego Health Sciences, La Jolla, CA USA; 2 Department of Anesthesiology, Perioperative and Pain Medicine, University of Calgary, Calgary, AB Canada; 3 VA San Diego Healthcare System, San Diego, CA USA

**Keywords:** Neuromodulation, Spinal cord stimulation, Parkinson’s disease, Gait, Salvage therapy

## Abstract

Parkinson’s disease (PD) is a progressive neurodegenerative disease with an incidence of 0.1 to 0.2% over the age of 40 and a prevalence of over 1 million people in North America. The most common symptoms include tremor, bradykinesia, rigidity, pain, and postural instability, with significant impact in quality of life and mortality. To date there is ongoing research to determine the optimum therapy for PD. In this review we analyze the current data in the use of spinal cord stimulation (SCS) therapy for treatment for Parkinsonian symptoms. We specifically address waveform pattern, anatomic location and the role of spinal cord stimulation (SCS) as a salvage therapy after deep brain stimulation (DBS) therapy. We also outline current experimental evidence from preclinical research highlighting possible mechanisms of beneficial effects of SCS in this context. Though the use of SCS therapy is in its infancy for treatment of PD, the data points to an exciting area for ongoing research and exploration with positive outcomes from both cervical and thoracic tonic and BURSTDR spinal cord stimulation.

## Background

Parkinson’s disease (PD) is a progressive neurodegenerative disease with an incidence of 0.1 to 0.2% over the age of 40 and a prevalence of over 1 million people in North America (Kalia and Lang 2015). The most common symptoms include tremor, bradykinesia, rigidity, pain, and postural instability, with significant impact in quality of life (Ha and Jankovic 2012; Martinez-Martin 2011) and mortality (Forsaa et al. 2010). A report of 618 patients with PD found that the transition from disease impairment to disability as defined by loss of independent function occurred generally between three and 7 years after the onset of PD (Shulman et al. 2008). Intervention targeting the impairments caused by PD is a crucial aspect of disease management.

The pathological mechanisms of the motor symptomology of PD center around the dysfunction of the substantia niagra pars compacta (SNc) and depletion of dopamine neurons. The reduction of dopamine in the nigrostriatal pathway to the caudate and putamen subsequently results in reduced inhibition of the thalamus and thus reduced excitatory input to the motor cortex, ultimately expressing as bradykinesia and other parkinsonian signs. Related to these physiological changes is the altered electrical communication within the nigrostriatal pathway. It was found that synchronized oscillatory activity at 10–35 Hz, as measured by deep brain electrodes, may mediate certain parkinsonian features and can be reduced by treatments using both dopamine agonists or by disruption of synchronized oscillatory impulses with direct electrical current stimulation (Gatev et al. 2006; Silberstein et al. 2005). As such, the primary modes of management of PD includes dopamine replacement therapy and bioelectric implantation using deep brain stimulation (DBS), which can directly disrupt the pathological synchronized oscillations.

Dopamine agonists are the gold standard for the treatment of PD. However, dopamine agonisms may be associated with loss of efficacy with prolonged use, necessitating increased dosing frequency, as well as issues with absorption (LeWitt et al. 2019). Invasive procedures like DBS have been utilized more recently with significant improvements in PD symptoms (Mills-Joseph et al. 2019; Okun 2012). DBS procedures are inherently moderate to high risk as they require cranial burr hole, carry a risk of infection, intracranial hemorrhage (up to 5.0%), seizures (up to 2.4%) and also may have diminished magnitude of improvement over time or failure after impantation (Okun 2012). The risk of infection has been reported to range from 1.2 to 15.2% (Okun 2012). Functional movement disorders can also arise after DBS, including involuntary movement of the extremities, weakness, and impaired balance (Breen et al. 2018). Failure may be related to lead migration, suboptimal patient selection, suboptimal therapy programming, disease progression, and/or development of tolerance or habituation (Okun 2012; Okun et al. 2008). In one retrospective study, misplaced leads had led to the majority of DBS failure (Okun et al. 2005). Additionally the treatment may only apply to a selective population of 1–4% of patients with PD, thus leaving large groups of patients without further treatment beyond standard conservative care (Morgante et al. 2007).

**Fig. 1:**
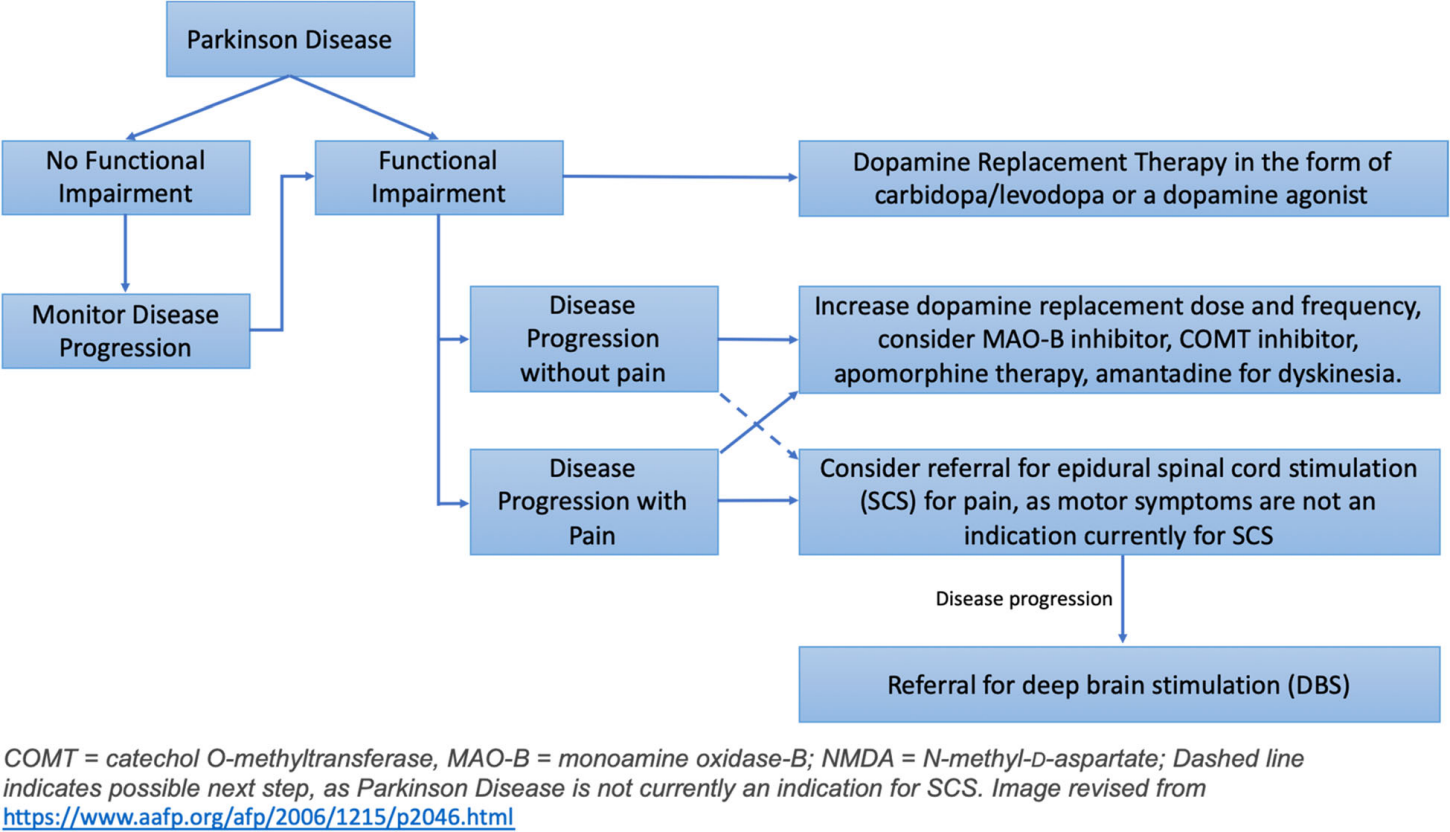
Potential Treatment Paradigm for Parkinson’s Disease using SCS and DBS

Another emerging electrical system that may disrupt the pathological neuronal oscillations in the basal ganglia in patients with PD is spinal cord stimulation (SCS) (Fuentes et al. 2009). Spinal cord stimulation of the dorsal columns within the epidural space is an emerging bioelectronic technology that has been extensively studied in multiple painful conditions (Caylor et al. 2019). More recently, SCS has been shown to improve locomotor symptoms in both animal models and human subjects with PD (Hassan et al. 2013; Santana et al. 2014). As previously mentioned, many patients with PD also have concurrent pain conditions that may also be responsive to the typical use of SCS (Fénelon et al. 2012). Interestingly, there is also a subgroup of patients whose SCS therapy was used as salvage therapy after loss of efficacy to both dopamine agonists medications and DBS, leading to the possibility that SCS may be a viable alternative or conjunctive therapy to DBS for the management of PD symptoms, as well as pain (Pinto de Souza et al. 2017) (Fig. 1). This article aims to summarize and discuss preclinical translational data for SCS in PD as well as clinical cases of SCS for PD as both singular bioelectric therapy and salvage therapy after loss of efficacy of DBS. Data sources for this relevant literature search included PubMed, MEDLINE/OVID, SCOPUS, and manual searches of the bibliographies of known primary and review articles with keywords Parkinson’s disease, spinal cord stimulation, and deep brain stimulation.

## Preclinical data and mechanisms

The mechanisms involved in the pathology of PD and subsequent clinical presentation are complex, involving multiple motor circuits and pathways within the nervous system (Davie 2008). The dysfunction of the SNc and depletion of dopamine within the striatum is integral to the majority of the motor symptoms seen in PD. A thorough understanding of the possible mechanisms for the pathophysiology related to PD is crucial in order to conceptualize and develop novel treatments, such as SCS.

Further research has sought to reveal specific neurophysiologic activity in relation to the pathology observed (Fuentes et al. 2010). Low-frequency synchronization in an oscillatory pattern within the basal ganglia is seen in both animal models and deep brain recordings of human patients with PD. The degree to which this contributes to clinically relevant motor symptoms is not well understood, as it is also observed in unaffected controls to varying degrees (Fuentes et al. 2010). Kuhn and colleagues found that the degree of excessive beta band (14–35 Hz) synchronization as measured by DBS in the subthalamic nucleus (STN) human subjects is correlated with the severity of bradykinesia and rigidity (Kühn et al. 2006). Furthermore, a reduction in the theta band synchronization via levodopa administration was reported with improvement in bradykinesia and rigidity. This link between dopaminergic activity and oscillations has been also thought to be a key to disrupting the normal physiologic output of the basal ganglia via the STN connection to the globus pallidus, SNr, and ﻿pedunculopontine nucleus (PPN) (Fuentes et al. 2010; Wichmann et al. 1994). While seemingly explanatory, the connection between the resting tremor seen in PD and pathologic oscillatory activity is not well established. In both patients with PD and animal models the correlation of resting trimotor to oscillatory activity is not consistently correlated (Rosin et al. 2007). It has been theorized that SCS may in fact disrupt this pathologic synchronous oscillatory activity via afferent input through the dorsal columns stimulated within the spinal cord leading to cortical ﻿desynchronization. Santana and colleagues examined SCS placed at the upper thoracic level in a primate model of PD (Santana et al. 2014). They found that SCS therapy lead to improvements in ﻿freezing, hypokinesia, postural instability, and bradykinesia. This was also strongly associated with desynchronization within the cortico-basal ganglia circuitry and reduction in beta-frequency oscillation.

Locomotion or gait in patients with PD can be severely affected and lead to serious injury due to falls (Davie 2008; Fuentes et al. 2009). The pathophysiology that leads to the abnormal gait in PD is not well elucidated at this time but is subject of ongoing research. While DBS has been shown to have significant benefit for dyskinetic motor symptoms of PD, akinesia, locomotion, and postural instability continue to be difficult to treat. The PPN within the midbrain has been theorized as a distinct entity from the SNc that is also affected in PD, leading to the hypokinetic symptoms observed (Jha et al. 2017). The PPN has been targeted via DBS and although needing further study, has been shown to improve gait and posture (Stefani et al. 2007). SCS alone has been shown to improve locomotion in animal models of PD by Fuentes and colleagues (Fuentes et al. 2009). Furthermore, SCS was combined with L-dopa at just one-fifth of the dose needed to otherwise create similar locomotive improvements, suggesting a synergistic effect. However, it is also likely the case that at least some dopamine is needed to be present for SCS to have an effect, as animals with < 1% of normal dopamine levels had no improvement with stimulation (Fuentes et al. 2009). In an effort to demonstrate the significance of spinal networks, Courtine and colleagues combined serotonergic agonists with epidural stimulation complete cord transection animal model with paralyzed rats, leading to weight-bearing treadmill locomotion (Courtine et al. 2009). Although, this locomotion was not voluntarily generated, the animal’s treadmill-based steppage was nonetheless indistinguishable from voluntary stepping. These patterned movements created at the level of the spinal cord in the absence of supraspinal input, suggests that locomotion itself can be affected via SCS.

### Cervical spinal cord stimulation

Clinically, SCS for PD has shown efficacy in several case reports and case series with leads placed both in the cervical and thoracic regions. One of the earliest of these was published in 2010 by Thevathasan et al. who based their cervical lead study off of previous animal model studies and reported two cases of patients with advanced PD treated by a single percutaneous electrode inserted at the top of C2 at both 130 Hz and 300 Hz (Thevathasan et al. 2010). After a 10-day follow-up, no significant change was found in VAS scores, 10-m walk, or UPDRS-III. Explanations for the negative results include lead localization of high cervical in the human patients and high thoracic in the rodent model suggesting that anatomical localization may play a role. In addition differences in electrodes relative to the size of the subject may have also played a role (Fuentes et al. 2009). However, other case reports of SCS implant at C2 have reported to result in functional improvement. One such report was of a PD patient who had chronic neck and upper extremity neuropathic pain treated who did well with medical management for 4 years until symptoms worsened. After SCS implant, symptoms not only improved immediately after implant but also continued to improve over time, from a UPDRS motor score of 28 at the early postoperative phase to a score of 22 at 1 year post-operatively. Interestingly, prior to permanent implant of the SCS, when trial leads were removed, pain returned immediately while PD symptoms gradually returned after 2 days (Hassan et al. 2013). It is unclear why this phenomenon of delayed return of motor symptoms occurred, but in animal models of PD with dorsal epidural stimulation of 30 s every 10 min, SCS not only alleviated hypokinesia during stimulation but also caused an increase in locomotion for 100 s after the stimulation period (Fuentes et al. 2009). It was postulated that SCS may recruit brainstem arousal systems and also promote depolarization and facilitate activation of striatal projection neurons that may explain this observed phenomenon.

Most recently, Mazzone et al. also noted SCS at C2–3 improved function in PD patients and also compared waveforms of BurstDR versus tonic stimulation (Mazzone et al. 2019). In this non-industry sponsored study, the primary indication for SCS was for pain in the tonic stimulation group and for parkinsonian motor symptoms in the BurstDR group. Three of the Burst group of patients had PD symptoms refractory to DBS while the other 9 patients were not DBS candidates. In comparing the two stimulation patterns, the authors found that a longer latency was needed prior to seeing benefits of motor changes in tonic stimulation as UPDRS-III scores were not significantly different in the acute post-SCS phase but was different in the 3, 6, and 12 months follow up data. Although both waveforms showed minor decrease of effectiveness for pain and motor control, the burst waveform showed attenuated decrease. In addition, electrical reprograming was required on average 17.6 ± 5.7 times in 3 months for the tonic group versus the 3.9 ± 0.9 for the burst group. These findings of delayed onset and frequent reprogramming requirements could explain potential loss of efficacy in certain cases (Table 1). Of note, a slight decrease of effectiveness for pain and motor control was observed 12 months after SCS implantation for both waveforms, and it is possible that similar to dopamine agonists and DBS, the motor effects of dorsal column stimulation may also be faced with decreased efficacy over time, but further longitudinal studies to validate and study this phenomenon is required.

**Table 1:** Cervical spinal cord stimulation data

Author & Article	N	Avg Age	Avg PD duration	Indication for SCS	DBS	Lead Location	Frequency; pulse width	Follow up period	Pain Scale Pre ➔ Post	Gait	UPDRS—III (Motor Exam)	Additional Comments
Thevathasan et al. 2010	2	76 ± 1.4	NA	Advanced PD	No	C2	130 Hz–300 Hz; 240–200 μsec	10 days	VAS Subthreshold *P* = 0.35 Suprathreshold *P* = 0.04	10 m walk *P* = 0.72	*P* = 0.44	Patients stimulated at two frequencies during with- and without- paresthesia conditions which was adjusted by increasing the amplitude of stimulation. Frequency for patient one was 130 Hz, which was chosen due to its use in DBS; frequency for patient two was 300 Hz, which was chosen due to animal studies. Measurements were done > 20 min after switching stimulation conditions, a time frame chosen based on subthalamic stimulation. No difference was found in function. The authors postulated that the lack of effect may have been due to type of stimulation as the animal model previous showed result with 30–60s intermittent bursts. Criticisms also fell on location of electrodes which were thoracic in the animal model.
Hassan et al. 2013	1	43	8	Neck and upper extremity pain	No	C2	40 Hz; 500 μsec	24 months	VAS 8 or 9 ➔ 0–2	10 m walk PostOp- 17 s 1 year - NA 2 year - 11 s	Post Op − 28 1 year - 22 2 year - 16	Patient did well with medical management for 4 years until symptoms worsened; SCS implant resulted in improved pain and PD motor symptoms. When trial leads were removed, pain returned immediately while PD symptoms gradually returned gradually in 2 days. Remarkably, after permanent implant at year two of follow-up, patient demonstrated improved motor scores compared to year one, although it may be possible that improvement in function lead to improved physical conditioning. Subjectively, she noted improved tremor and rigidity.
Mazzone et al. 2019	6	71 ± 7.3	17.1 ± 6.1	Back pain, vascular pain	No	C2–3	Tonic (135–185 Hz, 60–210 μsec)	12 months	VAS Improved (*P* < 0.05)	Gait speed *P* < 0.05 Cadence *P* < 0.05 Step length *P* < 0.05 Stride length *P* > 0.05	Tonic *P* < 0.05	The primary indication for SCS was for pain in the tonic stimulation group and for parkinsonian motor symptoms in the Burst group. The Burst group patient population were those who were deemed unsuitable for DBS except for 3 patients whose symptoms were refractory to DBS. The authors found that a longer latency was needed prior to seeing benefits of motor changes in tonic stimulation as UPDRS-III scores were not significantly different in the acute post-SCS phase but was different in the 3, 6, and 12 months follow up data. A slight decrease of effectiveness for pain and motor control was observed 12 months after SCS implantation for both waveforms, but burst waveform showed attenuated decrease. At the end of the 12 month study, L-dopa dose was reduced up for both groups (Tonic 1333.3 ± 471.9 mg to 1083.3 ± 2640 mg per day; Burst 835.0 ± 310.1 mg to 730 ± 273.7 mg). The study was funded by University grants and no industry sponsorship was indicated.
	12	65.5 ± 11.1	11.1 ± 5.3	PD or atypical parkinsonism	DBS in 3 cases	C2–3	Burst (250–500 Hz on; 40 Hz off, 1000 μsec)	12 months	VAS Improved (*P* < 0.05)	Gait speed *P* < 0.05 Cadence *P* < 0.05 Step length *P* < 0.05 Stride length *P* < 0.05	Burst *P* < 0.001	

Headings: *PD* Parkinson Disease, *DBS* deep brain stimulation, *Pain Pre ➔ Post* before SCS implant ➔ pain at the end of the reported follow up time, *TUG* Timed up and go, *UPDRS* Unified Parkinson disease rating scale. Other abbreviations: *Hz* Hertz, *μsec* microseconds, *m* meters, *VAS* visual analog scale

### Thoracic spinal cord stimulation (Table 2)

Studies of thoracic stimulation showed similar results of functional improvement. Fenelon et al. reported on a patient with PD who was treated with SCS at T9–10 for post laminectomy pain syndrome (Fénelon et al. 2012). The patient was followed for 29 months, and examinations were performed while SCS was switched on or off for 30–60 min at 100–130 Hz while the patient was on and off of dopamine medications. When SCS was switched on, UPDRS motor scores were reduced by 50% in the off-drug condition; surface EMG showed amplitude reduction but demonstrated no change in tremor frequency or pattern. More recently, Kobyashi et al. concluded from a two-week non-industry sponsored case report that BurstDR improved LBP, gait, and stooping posture (Kobayashi et al. 2018). Two weeks after BurstDR stimulation started at T6–8 (40 Hz burst with 5 spikes of 500 Hz), the patient showed improved reported pain as measured by SF-MPQ-2 and mental health measured by SF36, and the authors related this to the postulated mechanism of BurstDR in both the lateral discriminatory pain system and the medial affective pain experience. Fenelon et al. claimed BurstDR improved pain and motor function as well as tonic stimulation, however, it is unclear in the study if the author’s comparison of burst to tonic stimulation is in reference to previously reported cases or if the patient served as his own control with tonic stimulation first.

**Table 2:** Thoracic spinal cord stimulation data

Author & Article	N	Avg Age	Avg PD duration	Indication for SCS	DBS	Lead Location	Frequency; pulse width	Follow up period	Pain Scale Pre ➔ Post	Gait	UPDRS—III (Motor Exam)	Additional Comments
Fénelon et al. 2012	1	74	5	FBSS	No	T9–10	100–130 Hz 410 μsec	29 months	VAS off drug 6.9 ± 1.0 ➔ 1.9 ± 0.2	7 m walk and back. Off drug 29.3 ± 2.3 s ➔ 23.0 ± 6.3 s	Off drug 56.7 ± 3.3 ➔ 29.7 ± 2.5	All 4 examinations were performed while SCS was switched on or off for 30–60 min and the reported number is the average of the 4 examinations. Surface EMG showed amplitude reduction but no change in tremor frequency or pattern.
Agari and Date 2012	15	71.1 (range 63–79)	17.2 (range 7–39)	Low back and/or lower extremity pain	DBS in 7 cases	T7–12	5–20 Hz, 210–330 μsec	12 months	VAS 8.9 (range 7.8–10) ➔ 2.3 (range 0–3.3)	TUG 3 mo *P* < 0.01 1 year *P* > 0.05 10 m walk 3 mo *P* < 0.01 1 year *P* < 0.05	3 month *P* < 0.05 1 year *P* > 0.05	Large series of 15 patients with advanced Parkinson’s disease with 7 patients having DBS. Follow-up was 1 year and patients showed significant improvement in pain level and gait. Motor performance was significantly improved at 3 months but not at 1 year per UDPRS-III.
Nishioka and Nakajima 2015	3	74.3 ± 6.7	9.3± 4.0	Back Pain & leg pain	No	T8-L1	5–65 Hz 420–450 μsec	12 months	VAS 8.7 ± 1.5➔ 3.7 ± 0.6 *P* = 0.04	None	37.0 ± 5.3 ➔ 24.7 ± 5.8 *P* = 0.03	At 1 year follow up, SCS led to amelioration of chronic refractory pain and PD symptoms such as rigidity and tremor (scores based on UPDRS). Mental status did not significantly improve per MMSE (*p* = 0.19), and gait was not examined.
Kobayashi et al. 2018	1	74 M	3	Back Pain	No	T6–8	BurstDR 40 Hz with five spikes of 500 Hz burst; 1000 μsec	2 weeks	SF-MPQ 47 ➔ 18	None	20 to 6	BurstDR improved LBP, gait, and stooping posture. Patient showed improvement of low back pain and parkinsonism as well as mental health measured by Short-Form 36 (SF36 27.7 pre-SCS to 49.1 post-SCS), which was postulated to be related to the mechanism of BurstDR in both the lateral discriminatory pain system and the medial affective pain experience. Author described no financial disclosures.
Samotus et al. 2018	5	71.2 ± 9.8	14 ± 3.7	Parkinson Disease	No	T8–10	30–130 Hz, 300–400 μsec	6 months	NA	Step length *P* > 0.16 Mean stride velocity *P* = 0.05 sit-to-stand *P* = 0.04	32 ± 11.7 ➔ 21.4 ± 10.8 (*P* = 0.02)	Patients with freezing of gait underwent SCS for PD; pain state not noted. Spinal cord stimulation combinations (200–500 μs/30–130 Hz) at suprathreshold intensity were tested and it was found that setting combinations of 300–400 μs/30–130 Hz provided gait improvements. In addition to step length and motor score improvements, the mean number of freezing-of-gait episodes reduced significantly from 14.8 ± 15.4 pre-SCS to 0.2 ± 1.7 at 6 months post-SCS. Three of the 5 patients also required a mean reduction of daily levodopa by 115 mg by 6 months due to dyskinesias which were presumed to be due to dopamine excess.

Headings: *PD* Parkinson Disease, *DBS* deep brain stimulation, *Pain Pre ➔ Post* before SCS implant ➔ pain at the end of the reported follow up time, *TUG* Timed up and go, *UPDRS* Unified Parkinson disease rating scale. Other abbreviations: *Hz* Hertz, *μsec* microseconds, *m* meters, *VAS* visual analog scale, *SF-MPQ* short form McGill Pain Questionnaire, *SF36* short form 36 for quality of life, *MMSE* mini-mental status exam

Outside of single case reports, Nishioka and Nakajima reported 3 cases of PD patients who received SCS for back and leg pain implanted at T8–11 (freq 5–65 Hz) who at 1 year follow-up had decreased pain, rigidity, and tremor, and increase in UPDRS-III scores (Nishioka and Nakajima 2015). Mental status via the mini-mental status exam (MMSE) was used and no significance was found in cognitive function after 12 months. Samotus, Parrent, and Jog analyzed the effect of SCS in 5 PD patients with the difficult-to-treat freezing of gait in the absence of pain (Samotus et al. 2018). The authors tested SCS programing combinations (200–500 μs/30–130 Hz) at suprathreshold intensity, and it was found that setting combinations of 300–400 μs/30–130 Hz provided gait improvements. Motor improvements were measured as UPDRS-III scores and improved from 32 to 21. Gait was measured in step length, stride velocity, and time from sit-to-stand and improved 38–50% in all parameters after 6 months. In addition, the mean number of freezing-of-gait episodes reduced significantly from 14.8 ± 15.4 pre-SCS to 0.2 ± 1.7 at 6 months post-SCS. Three of the 5 patients also required a mean reduction of daily levodopa by 115 mg by 6 months due to dyskinesias which were presumed to be due to dopamine excess. On a larger scale, 15 patients with advanced PD with mean disease duration of 17.2 years who received SCS implants at T7–12 (Freq 5–20 Hz) for back, trunk, and/or leg pain showed significant improvement in pain level and gait at 1 year follow-up. Motor performance was significantly improved at 3 months but not at 1 year per UDPRS-III (Landi et al. 2013). On a larger scale, 15 patients with advanced PD (5 men and 10 women) with disease duration of 7–31 years who received SCS implants at T7–12 (Freq 5–20 Hz) for back, trunk, and/or leg pain showed significant improvement in pain level and gait at 1 year follow up. Motor performance was significantly improved at 3 months but not at 1 year per UDPRS-III (Agari and Date 2012).

### SCS as salvage for failed DBS therapy (Table 3)

It is also interesting that patients who initially do well with deep brain stimulation for PD and have decreased efficacy of the DBS over the years do well with SCS posing an important use of spinal cord stimulation as possible salvage therapy for failed DBS (Agari and Date 2012). Landi et al. reported a 65 year old female with previous DBS who received paddle leads at T9–10 for lower extremity pain showed improvement in both pain and gait (Landi et al. 2013). Her time to cover a length of 20 m decreased 20% during stimulation. UPDRS III was unchanged after SCS paddle placement. Subjective evaluation of quality of life (EQ-VAS) improved 60%. Similarly, 65-year-old who had previously done well with carbidopa/levodopa, cabergoline, and deep brain stimulation underwent SCS for painful camptocormia, an anterior flexion of the thoracolumbar spine that exists while upright but disappears in the supine position. It was noted that 1 year after commencing DBS, camptocormia had disappeared completely but then reappeared at 62 years of age which prompted SCS for pain (Akiyama et al. 2017). After SCS implant, motor function improved in terms of TUG which was 15 s pre-SCS and 7 s post-SCS at day 29. UPDRS-III did not change but it was noted that UPDRS-II (ADL based) significantly improved from 25 pre-SCS to 10 post-SCS at day 11 and a score of 12 at day 29. Camptocormia was also noted to improve. In a case series of 4 PD patients with DBS to the STN, paddle leads at T2–4 lead to improvement in TUG, 20 min walk, UPDRS-III and PDQ39. In this study, locomotion and gait were recorded at baseline, as well as 1, 3, and 6 months after SCS at both 60 Hz and 300 Hz and with normal use of their DBS. Improvement occurred within minutes after stimulation onset and lasted for 6 months. Gait improvement was only documented with SCS was delivered at 300 Hz. At 6 months, TUG scores improved by 63% (*P* = 0.006) while the 20 m walk time was reduced by 58% and number of steps reduced by 65.7% (*P* = 0.05 and 0.009, respectively). Stride length increased by 170% (*p* = 0.01). PDQ39 improved by 44.7% (from 58 to 32, *p* = 0.002), UPDRS III was 36.3% at 6 months (15, *P* = 0.034) (Pinto de Souza et al. 2017).

**Table 3:** Salvage spinal cord stimulation data in patients with loss of efficacy to deep brain stimulation

Author & Article	N	Avg Age	Avg PD duration	Indication for SCS	DBS	Lead Location	Frequency; pulse width	Follow up period	Pain Scale Pre ➔ Post	Gait	UPDRS—III (Motor Exam)	Additional Comments
Agari and Date 2012^a^	15	71.1 (range 63–79)	17.2 (range 7–39)	Low back and/or lower extremity pain	DBS in 7 cases	T7–12	5–20 Hz, 210–330 μsec	12 months	VAS 8.9 (range 7.8–10) ➔ 2.3 (range 0–3.3)	TUG 3 mo *P* < 0.01 1 year *P* > 0.05 10 m walk 3 mo *P* < 0.01 1 year *P* < 0.05	3 month *P* < 0.05 1 year no change	Large series of 15 patients with advanced Parkinson’s disease with 7 patients having DBS. No subgroup analysis was performed for only the DBS patients. Follow-up was 1 year and patients showed significant improvement in pain level and gait. Motor performance was significantly improved at 3 months but not at 1 year per UDPRS-III.
Landi et al. 2013	1	65	8	Leg pain	DBS	T9–10	30 Hz, 250 μsec	16 months	VAS Improved up to 70%	Time to 20 m walk Decreased 20%	No change	Patient with DBS demonstrated improved walking speed after SCS and did not need assistance to walk, although it is unclear the degree of assistance necessary to ambulate prior to stimulation. UPDRS III on versus off condition was unchanged after SCS surgery. Subjective evaluation of quality of life (EQ-VAS) also improved 60%.
Pinto de Souza et al. 2017	4	64.25 ± 5.91	21.25 ± 10.18	Advanced PD	DBS	T2–4	300 Hz 90 μsec	6 months	–	TUG: *P* = 0.006 20 m walk: *P* = 0.02 Steps in 20 m walk: *P* = 0.009	*P* = 0.03	Improvement in locomotion occurred within minutes after stimulation onset and lasted for duration of study with no apparent loss of benefit over time. Patients were kept on their normal DBS settings during the study. To deter placebo effect of open label design and patient reported stimulation-induced paresthesia, blinded experience where SCS was randomly delivered at either 60 or 300 Hz; despite similar paresthesia, gate improvement was only documented with SCS was delivered at 300 Hz.
Akiyama et al. 2017	1	65	12	Back pain	DBS	T8	Program 1: 7 Hz, 450 μsec Program 2: 7 Hz, 250 μsec	1 month	VAS 10 ➔ 2 (post op day 1)	TUG Pre 15 s Post 7 s	No change	Patient who had previously done well with carbidopa/levodopa, cabergoline, and deep brain stimulation underwent SCS for painful camptocormia with Pisa. It was noted that 1 year after commencing DBS, camptocormia had disappeared completely but then reappeared at 2 years after commencing DBS which prompted SCS for pain. After SCS implant, TUG improved, and although UPDRS-III did not change, UDPRS-II (based on activities of daily living) significantly improved from 25 pre-SCS to 12 at day 29. Camptocormia was also noted to improve as measured by angles of forward flexion from the vertical axis.
Mazzone et al. 2019^a^	12	65.5 ± 11.1	11.1 11.1 ± 5.3	PD or atypical parkinsonism	DBS in 3 cases	C2–3	Burst (250–500 Hz on; 40 Hz off, 1000 μsec)	12 months	VAS Improved (*P* < 0.05)	Gait speed *P* < 0.05 Cadence *P* < 0.05 Step length *P* < 0.05 Stride length *P* < 0.05	Burst *P* < 0.001	See Table 1 for additional group of non-DBS patients. There were 3 patients refractory to DBS who received Burst stimulation. No subgroup analysis was performed for only the DBS patients. The authors found differences motor scores, gait, and pain in the post-implant acute, 3, 6, and 12 months follow up data. Overall in the Burst group, L-dopa therapy was reduced 835.0 ± 310.1 mg to 730 ± 273.7 mg per day.

Headings: *PD* Parkinson Disease, *DBS* deep brain stimulation, *Pain Pre ➔ Post* before SCS implant ➔ pain at the end of the reported follow up time, *TUG* Timed up and go, *UPDRS* Unified Parkinson disease rating scale. Other abbreviations: *Hz* Hertz, *μsec* microseconds, *m* meters, *VAS* visual analog scale

^a^Data also seen in Table 1 or Table 2; added to Table 3 in order to be inclusive of all DBS patients

## Conclusion

Motor symptoms and pain in PD can impact quality of life, and lead to disability as well as mortality. Current management includes dopamine therapy and DBS each with its own challenges and decreased efficacy with prolonged use. However, in recent years, it has been demonstrated that SCS for PD can be used as both a singular bioelectric therapy and salvage therapy after loss of efficacy of DBS, although the mechanisms remain shrouded in mystery. It may be possible that electrical stimulation of spinal cord sends signals to basal ganglia circuits which then in turn increases release of stored dopamine similar to DBS in pigs (Shon et al. 2010). There may also be a neuroprotective component achieved by electrical stimulation that delays progression of dopaminergic neuron loss in the brain. It is also of note that in combination with SCS, a decreased dose of L- DOPA was enough to produce equivalent locomotion to L-dopa alone in the rat model. A better understanding of how to optimally combine dopamine replacement therapy and electrical stimulation will be a very important future goal in order to develop better strategies to alleviate motor symptoms in PD.

As previously mentioned, the study by Thevathasan et al. showed that SCS failed to relieve akinesia or restore locomotion in PD when leads were placed in the high cervical position, while a recent case report by Fenelon et al. showed SCS was able to improve abnormal posture and gait disorders when the leads were placed at the T9-T10 level (Fénelon et al. 2012). This variability in data has led to a demand for more studies to definitively conclude if SCS has an improved role compared to DBS in PD patients, and if these modes of neuromodulation could perhaps act synergistically. There remains a paucity of data on the potential synergistic effects of SCS and DBS in PD patients with regards to improvements in gait and postural instability. Certainly, the neuroanatomy of gait function involves all levels of the nervous system, and it can be difficult to pinpoint which single specific area would benefit most from stimulation to improve gait function. The synergistic effects of SCS and DBS offer a neuromodulatory approach capable of stimulating multiple complementary neuronal areas in gait and postural function and optimizing transmission in spinal locomotor tracts.

Different stimulation patterns and frequencies have been considered when determining efficacy. In the Mazzone et al. study that compared tonic stimulation versus burst stimulation in the high cervical region (C1–2 or C2–3), patients programmed with the burst mode of stimulation showed faster onset of motor improvement as well as required fewer adjustments to programming in a 3-month period. More research is needed to determine maximum efficacy at specific spinal levels as well as mode of stimulation. There is one clinical trial listed in www.clinicaltrials.gov for a future study on the effects of SCS on freezing of gait in patients with PD (NCT03526991).

## Data Availability

Not applicable. If your manuscript does not contain any data, please state not applicable in this section.
